# All That Glitters Is Not Gold: The Other Insects That Fall into the Asian Yellow-Legged Hornet *Vespa velutina* ‘Specific’ Traps

**DOI:** 10.3390/biology10050448

**Published:** 2021-05-20

**Authors:** Omar Sánchez, Andrés Arias

**Affiliations:** Department of Organisms and Systems Biology (Zoology), University of Oviedo, 33071 Oviedo, Spain; omarelrdd@hotmail.com

**Keywords:** invasive species, exotic species, non-target insects, services, disservices, *Varroa*, biodiversity, Iberian Peninsula, Spain, taxonomy

## Abstract

**Simple Summary:**

The recent spreading of the invasive Asian hornet (*Vespa velutina*) to the Iberian Peninsula has led to the application of management measures to control and mitigate its impact on receiving environments. Among the most used control methods are capture traps, which use a sugary attractant to catch the invasive wasps. However, although the used *V. velutina* traps are presumably specific, they do not only attract *V. velutina* specimens, but also a large number of non-target species that are also captured. In the present work, the species of insects that unintentionally fall into the capture traps of *V. velutina* have been specifically identified, as well as their implications for ecosystem and for human activities. A total of 74 non-target taxa of insects were caught by the *V. velutina* trapping in northern Spain. Most of them were flies, mosquitoes, wasps and moths, being all highly important groups from the biological, ecological and economical points of view. Surprisingly, the most abundant trapped species was the invasive fly, *Drosophila suzukii* that represented the 36.07% of the total catches. Furthermore, we reported the first record of ectoparasitic mites of the genus *Varroa* on *V. velutina*, constituting a newly recorded symbiotic association.

**Abstract:**

The introduction of invasive species is considered one of the major threats to the biodiversity conservation worldwide. In recent years, an Asian invasive species of wasp has set off alarms in Europe and elsewhere in the world, *Vespa velutina*. The Asian wasp was accidentally introduced in France around 2004 and shortly thereafter it was able to colonise practically all of Europe, including the Iberian Peninsula. The ecological and economic implications of *V. velutina* invasion and its high colonisation ability have triggered widespread trapping campaigns, usually supported by beekeepers and local governments, with the aim of diminishing its population and its negative impacts. Among the most used control methods are the capture traps, which use a sugary attractant to catch the invasive wasps. However, the species-specific selectivity and efficiency of these traps has been little studied. In this paper, we have analysed the specific identity of the unintentionally trapped insect species from northern Spain (covering one-year period), as well as we have assessed the provided ecosystem services by them. A total of 74 non-target taxa of insects were caught by the *V. velutina* studied traps, most of them correspond to the orders Diptera, Hymenoptera and Lepidoptera, the dipterans being the most abundant group. Surprisingly, the most abundant trapped species was the invasive fly, *Drosophila suzukii* that represented the 36.07% of the total catches. Furthermore, we reported the first record of ectoparasitic mites of the genus *Varroa* on *V. velutina*, constituting a newly recorded symbiotic association. Hopefully, the provided information helps to develop new protocols and management tools to control this invasive species in the Iberian Peninsula and other temperate areas of western Europe and the Mediterranean basin.

## 1. Introduction

Ongoing human activities, such as agricultural intensification and associated land use changes, habitat destruction and fragmentation, global warming and the spreading of invasive species are causing extensive shifts in native biodiversity worldwide [1,2,3,4,5,6,7,8,9,10]. Biological invasions constitute a multiple threat for biodiversity, economic activities, and even the human health. In recent years, an invasive species of wasp—*Vespa velutina* Lepeletier, 1836—has set off alarms in Europe and elsewhere in the world [1,2,3,4,5,6,7]. This famous species, commonly known as yellow-legged wasp or Asian wasp, is native to eastern Asia and belongs to the family Vespidae. *Vespa velutina* was accidentally introduced with ceramic boxes from China into Europe around 2004, where it was firstly detected in the French area of Lot-et-Garonne [1,2]. Since its successful introduction in France, it has rapidly colonised other European countries such as Spain, Portugal, Belgium, Italy, the United Kingdom, The Netherlands and Germany. Its rapid dispersal has been explained by the fact that *V. velutina* has not enough autochthonous direct competitors or predators; the local inexhaustible food sources; its high reproduction rates, and the European climate conditions that favour its proliferation and spread into new areas [3,4].

*Vespa velutina* was detected for the first time in Spain in 2010 in Amaiur (Navarra, northern Spain) and since then it was able to colonize practically the whole northern half of Spain, from Galicia to Catalonia [5,6]. The Asian wasp is considered as a threat to current biodiversity. It is a generalist predator of medium-sized insects like other Hymenoptera and Diptera, showing a special predilection for the European honeybee (*Apis mellifera* Linnaeus, 1758) [1,2]. After its rapid and successful invasion, the Asian wasp has exterminated entire beehives of the *A. mellifera*, decreasing thereby the availability and supply of apiculture products and causing substantial losses in this economic sector [7]. Also, *V. velutina* outcompetes with the autochthonous European hornet *V. crabro* Linnaeus, 1758 for the same ecological niche and food sources with consequent ecological repercussion [11,12]. On the other hand, like other vespids, their bite can cause serious health problems for allergy sufferers [12]. Its invasion has triggered important socio-economic impacts [7,13] and thus, the Asian wasp was officially considered as an invasive species and was included in the Spanish Catalogue of Invasive Species (Catálogo Español de Especies Invasoras, R D 630/2013). Furthermore, it is listed as an invasive alien species of Union concern (EU Regulation 1141/2016) in the framework of the respective European regulation (EU Regulation 1143/2014). After the arrival of the Asian wasp in Europe, serious concern has arisen as to the real impact on local insect populations due to its predatory nature [14], especially after the knowledge about its predilection to prey on European honeybees [1,2].

Consequently, government administrations of several European countries are monitoring the spreading dynamics of *V. velutina*, aiming to detect their early presence on newly colonised areas and to implement control strategies to prevent/reduce their expansion. Currently, the main control strategies are based on trapping adults, using different types of traps and baits, and nest detection and destruction [13,14,15,16,17]. Paradoxically, there are few studies on the efficiency and selectiveness of these traps and their attractants on the Asian hornet [18,19]. It is known (although not species-specifically quantified) that the currently utilized traps do not only attract *V. velutina* specimens, but also other species of insects, such as other hymenopterans (bees, wasps and allies) and members of the order Diptera (flies and mosquitoes) [1,2,13,18,19]. Furthermore, the proportion of these non-target insects in *V. velutina* traps stress the need of developing alternative monitoring and control techniques [18]. Dipteran and hymenopteran species are essential for the proper ecosystem functioning, as well as for being providers of great benefits to the agriculture (e.g., the production of entomophilous crops) and other human activities [18,19,20,21]. However, as a consequence of the little published/available information on this regard, the potential impacts of catches of certain groups of insects (e.g., the pollinators) cannot be properly assessed. Thus, it is crucial to know the specific identity of the entomofauna affected by the *V. velutina* traps, as well as the functional roles that they play in the ecosystem.

The main goal of this work is to assess the specific diversity of the non-target insect species captured by *V. velutina* traps, taking the Principado de Asturias (northern Spain) as a study case. This study aims as well to assess the functional roles of the trapped insects (ecosystem services and disservices) in the ecosystem and their status (native, exotic, invasive), in order to achieve a better understanding of the real effects of *V. velutina* traps in the ecosystem. Hopefully, this information helps to develop new protocols and management tools to control this invasive species in the Iberian Peninsula and other temperate areas of western Europe and the Mediterranean basin.

## 2. Materials and Methods

**Figure 1:**
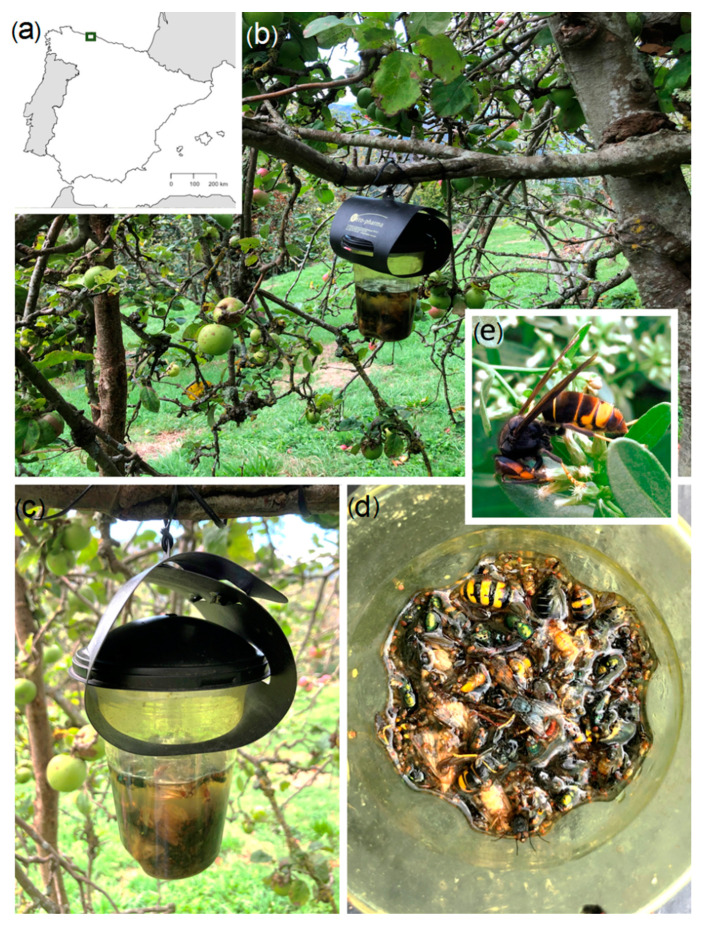
Location of the sampling traps in Asturias (northern Spain) (**a**); General view of the plot (**b**); detail of “Vespa Catch” type trap placed on the branch of an apple tree in the study location (**c**); sample of captured insects in one of the studied traps (**d**); *Vespa velutina* specimen from the study site (**e**).

Diverse environmental factors (i.e., land cover, habitat structure, or human disturbance) can influence the effectiveness of the baited traps, increasing the chances to capture *V. velutina* in particular areas, such as rural localities where the governmental trapping campaigns were carried out in northern Spain (see https://www.asturias.es/general/-/categories/613173?p_r_p_categoryId=613173 accessed on 4 May 2021). Thus, for the study of the diversity of insects unintentionally captured by *V. velutina* traps, we analysed eight monitoring traps located in a plot with a majority plantation of apple trees located in a rural area of Loredo, Asturias, northern Spain (43°24′ N–6°44′ W, 310 m altitude) (Figure 1). At this locality, *V. velutina* (Figure 1e) has been present since 2016.

The trap type used was the VespaCatch^®^ trap made by Véto-pharma (Palaiseau, France, Figure 1b,c) and the bait was VespaCatch^®^ attractant made also by Véto-pharma (for additional information see https://www.blog-veto-pharma.com/ accessed on 4 May 2021). We selected this trap type and attractant because they were the ones used by the Principado de Asturias Regional Government during the trapping campaigns that they carry out annually (see https://www.asturias.es/general/-/categories/613173?p_r_p_categoryId=613173 accessed on 1 May 2021). Traps were positioned on apple trees at a height of 1.5 m from the ground (Figure 1b), with a distance of 10 m between them. The traps covered one-year period, from April 2020 to March 2021. They were activated during four consecutive weeks (30 trapping days each) in four seasons: spring, summer, autumn and winter. The traps were checked every 4–5 days, at the same time, the attractant was renewed, and the trapped fauna (Figure 1d) were collected and temporarily stored in 70% ethanol until pinned for taxonomic identification. All collected specimens were identified to species level except for some lepidopteran specimens that had a high degree of deterioration and for the members of the families Phoridae, Sciaridae and Formicidae, which require molecular studies for the proper specific determination. For the specific identification, both external and internal (e.g., genitalia) morphological characters were examined under both dissecting stereomicroscope and compound light microscope. Selected specimens were photographed with a DFC310FX camera (Leica, Wetzlar, Germany) mounted on a Leica M205FA stereomicroscope. The specimens were identified by the authors and subsequently deposited at the Zoological Collection of the Department of Organisms and System (BOS) of the University of Oviedo (https://bos.uniovi.es/artropodos accessed on 1 May 2021).

For the community analysis, species abundance data were analysed, using PRIMER v. 6 community analysis software. To visualize the differences in species composition among seasons a matrix of similarity was constructed by means of Bray Curtis similarity coefficient [22], as well as a cluster (group-average mode) and a multidimensional scaling (MDS). SIMPER analysis was carried out to identify the species that characterized the different groups. The phenological study was carried out by plotting the abundances of the most common and representative species of each season to check biological patterns.

## 3. Results

### 3.1. Specific Diversity of the Non-Target Insect Species

**Figure 2:**
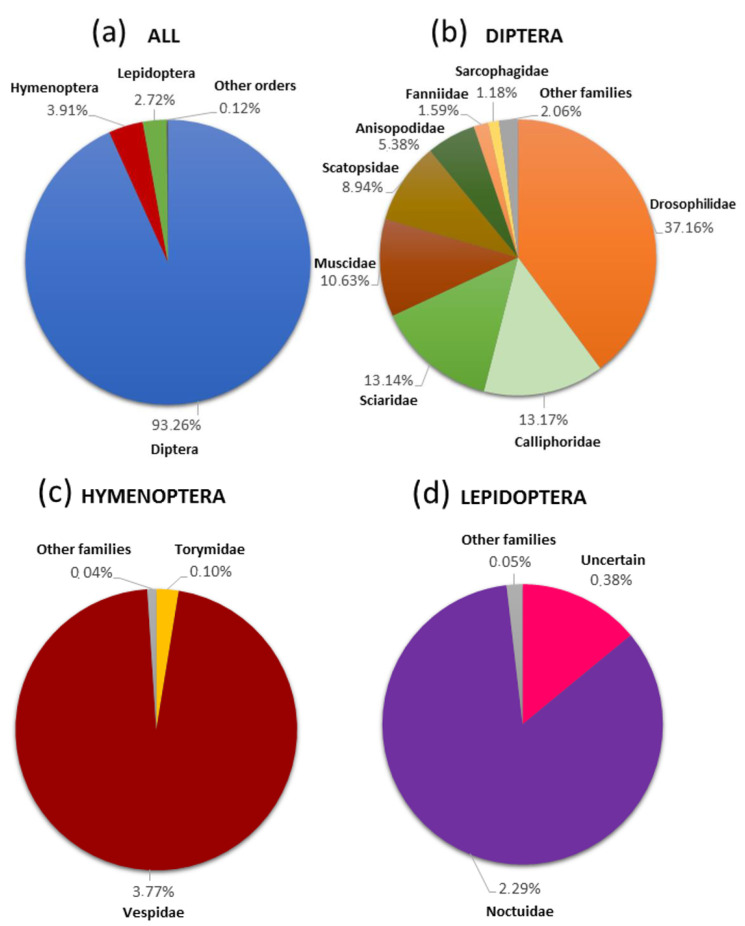
Percentages of the different orders of insects trapped in this study (**a**) and families most representative of the orders Diptera (**b**), Hymenoptera (**c**) and Lepidoptera (**d**).

A total of 12,835 trapped specimens were collected and examined. They belonged to 74 different taxa grouped into seven arthropod orders (Table 1; Figure 2a). The order Diptera was the most abundant in the traps, representing 93.26% of the total samples and harbouring 53 taxa (Figure 1b). Within dipterans the most common species caught was the invasive species *Drosophila suzukii* (Matsumura, 1931), explaining the 36.07% of the total catches. The order Hymenoptera was the second group most affected by traps, constituting 3.91% of the total and affecting 10 species (Figure 1c). Among hymenopterans, the percentage of *V. velutina* caught was 2.23% and one of its congener *V. crabro* Linnaeus, 1758 was 1.45%. The incidence of other hymenopteran species was anecdotal, representing only 0.23% of the total sample (Table 1). The trapped species of the genera *Vespula* and *Torymus* had abundance percentages of 0.09% and 0.1% respectively. The order Lepidoptera was the third group most affected, constituting 2.72% of the total and involving at least 5 taxa (some lepidopteran specimens cannot be identified due to their high degree of deterioration) (Figure 1d). In relation to the butterflies caught, we can highlight the presence of two invasive species, the true armyworm moth (*Mythimna unipuncta* (Haworth, 1809)) and the geranium bronze (*Cacyreus marshalli* Butler, 1897), whose abundances in the total sample were 2.29% and 0.03% respectively. 

**Figure 3:**
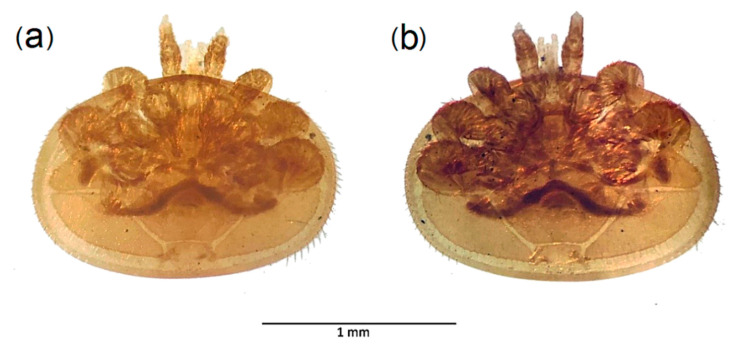
Micrographs of ethanol preserved specimens of *Varroa* found on *Vespa velutina*. Dorsal view of Varroa(**a**) and ventral view of same (**b**).

In addition to the mentioned insect species, three species of other taxonomic groups were found, i.e., *Pomatias elegans* (Müller, 1774) (Mollusca: Gastropoda: Pomatiidae), *Dicranopalpus ramosus* (Simon, 1909) (Arthropoda: Chelicerata: Arachnida: Opiliones) and a spider of the Family Salticidae (Arthropoda: Chelicerata: Arachnida: Araneae). Ectoparasitic mites of the genus *Varroa* (Arachnida: Acari: Parasitiformes) were found attached on five specimens of the 286 examined *V. velutina* specimens (one mite per *V. velutina* specimen). The prevalence rate of the *Varroa* parasite in *V. velutina* was of 0.017 (1.75%). In all cases, the mites were found clinging to the lateral-ventral part of the abdomen of *V. velutina* specimens. Additionally, two free specimens of the same mite genus were found in the attractant liquid from the traps. *Varroa* specimens found measured between 1.5 and 2 mm and corresponded with two colour morphotypes, one with darker colour (reddish-brown) and the other lighter (amber color) (Figure 3). The analysis of their morphological features showed that they were consistent with the diagnosis of the genus *Varroa*. This finding constitutes the first record of this genus of parasitic mites on *V. velutina*, presenting a newly recorded symbiotic association.

**Figure 4:**
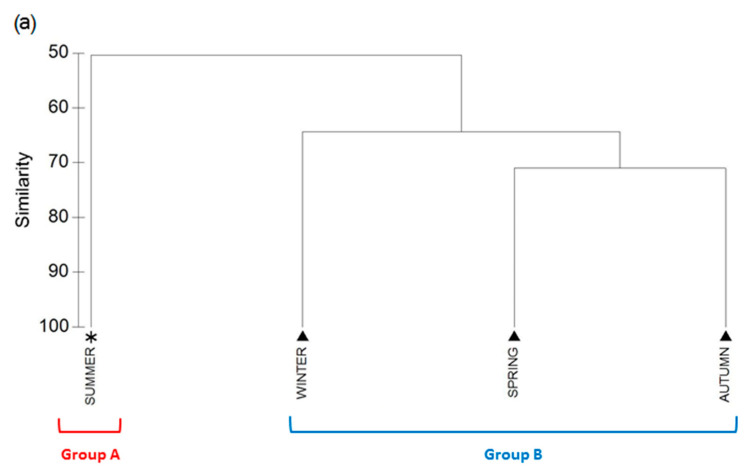

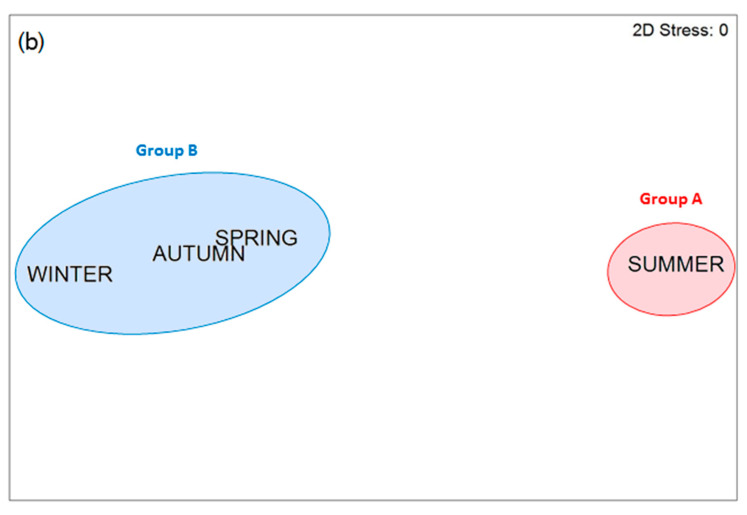
Cluster dendrogram (**a**) and MDS plot (**b**) based on the similarity of the insect’s abundance among seasons. Symbols identify the different assemblages as defined by branches in the dendrogram. Ellipses encapsulate all samples from the same assemblage in the MDS plot.

Cluster analysis carried out with abundance data showed two assemblages, Group A (Summer) and Group B (spring, autumn and winter) (Figure 4). Group A clustered the samples of summer and included 53 taxa. 

Group B gathered the samples from spring, autumn and winter, with an average similarity of 66.5%. The taxa richness was 67, being *D. suzukii* (Diptera) and Family Sciaridae (Diptera) the ones that mostly contribute with 37.59% and 16.93% respectively. The SIMPER analysis showed a 49.67% of dissimilarity between Group A and Group B. This is accounted for by the major representativeness of *D. suzukii* and *Lucilia caesar* (Diptera) with percentages of 50.35% and 8.83% respectively (Table A1).

### 3.2. Ecosystem Services and Disservices

**Figure 5:**
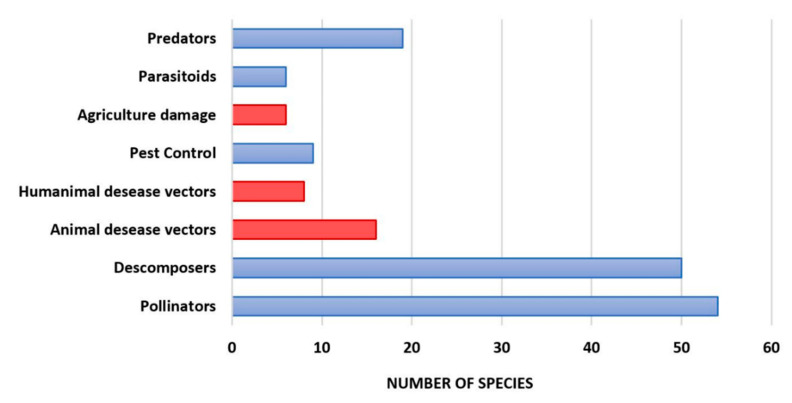
Number of species that perform the different ecosystem services (blue) and disservices (red) among those found in the studied sample.

The results obtained from the analysis of services and disservices showed that 73% and 67.6% of the trapped species were pollinators and decomposers, respectively. Predatory species (25.7%) were also compromised to a great extent. The proportion of species that produce disservices was quite high (Figure 5). 

Among them, we can highlight those that cause damage to agriculture (8.1%) and those that act as vectors of both animal (21.6%) and human (10.8%) diseases (Figure 5).

### 3.3. Phenological Study

**Figure 6:**
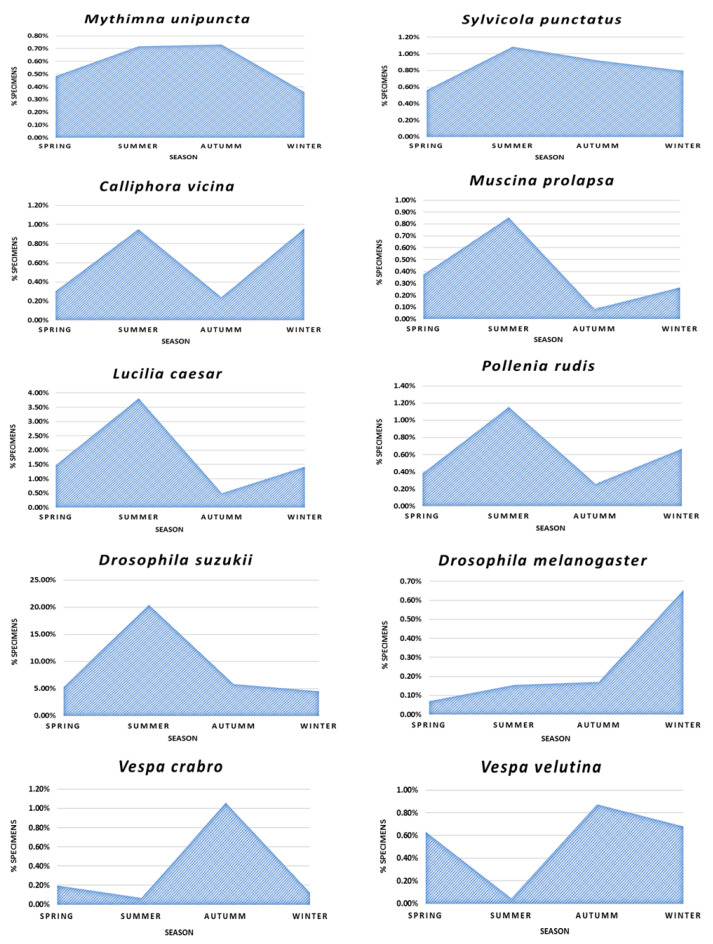
Phenological analysis of the most abundant and representative non-target species of the total sample during the four seasons of the year based on the percentage of the total number of individuals.

For the phenological analysis (Figure 6), the most abundant and representative species of the total sample were selected, i.e., seven species of Diptera (*L. caesar*, *Pollenia rudis*, *Calliphora vicina*, *Muscina prolapsa*, *D. suzukii*, *D. melanogaster* and *Sylvicola punctatus*), the lepidopteran *Mythimna unipuncta* and the hymenopterans *V. velutina* and *V. crabro*.

The presence of both species of the genus Vespa (*V. velutina* and *V. crabro*) were confirmed throughout the whole year, showing similar phenological cycles and an abundance maximum in autumn. *Vespa velutina* was also a slightly lower in spring (Figure 6). Queens of both species were only found during the spring season, the remaining specimen captures corresponded to workers and to a lesser extent to males.

The phenological analyses showed that *M. unipuncta* reach the maximun number of individuals in summer and autumn and the rest of the year remains in intermediate abundances. The species *L. caesar*, *P. rudis*, *C. vicina* and *M. prolapsa*, present very similar life cycles, reaching the maximum number of individuals in summer and falling drastically in autumn, apart from *S. punctatus*, whose number of individuals decreases in a more attenuated way, reaching its minimum in winter. However, *C. vicina*, seems to present a biannual cycle, presenting two maxima, one in summer and the other in spring. *Drosophila suzukii* present its maximum during the summer and reach very low abundances in autumn and winter. However, *D. melanogaster*, shows an inverse trend reaching its maximum in winter.

## 4. Discussion

The results of this study have shown that *V. velutina* traps, apart from its primary function (i.e., to control wasp populations), can be also used for detection of new invasions of insect pests and/or parasites, for delimitation of infestation areas and for monitoring population levels of established pests of non-target species. This information can be used to make decisions on the initiation of control measures or to measure effectiveness of a pest management program. In this study, the careful analysis of the traps has shown the occurrence of a new symbiotic relationship between *V. velutina* and *Varroa* mites. *Varroa jacobsoni* Oudemans, 1904 is a parasite that feeds on the hemolymph of bees of the genus Apis [23]. It was first described in Indonesia parasitising the Asian honeybee (*Apis cerana* Fabricius, 1793) [23,24], although it can also parasitise other hymenopterans such as wasps of the genus *Vespula* (Jeliński, 1990). In 2000, Anderson and Trueman [23] conducted a molecular study that showed that *V. jacobsoni* was actually a species complex and a new species was described, *V. destructor* Anderson & Trueman, 2000. Currently it is considered that *V. destructor* is mainly responsible for the damage to the beekeeping sector, since it is commonly found on European honeybees (*A. mellifera*) [23]. Furthermore, *V. destructor* acts as a vector for the wing strain virus (DWV) that can lead to the death of an entire colony of bees or hymenopterans [23]. Its role as a vector for another RNA virus, the Moku virus (MV), that affects wasps of the genus *Vespula* has recently been discovered [25]. Both viruses, DWS and MV, are generalists and may infect a wide variety of insects [25]. Thus, the relationship between *V. velutina* and *Varroa* should be studied further, since the Asian wasp could constitute a new dispersal vector of *Varroa* and its potential carried viruses.

On the other hand, our results have shown that the insect orders most affected by *V. velutina* trapping in northern Spain are Diptera, Hymenoptera, and Lepidoptera, confirming previous studies [18,19]. These three orders are very important groups in terms of the provided ecosystem services (Figure 5; Table 2). It is noteworthy that six dipteran families are of special interest from an economic point of view due to their agricultural, veterinary, or medical involvement [26,27]. Also, agronomically they are of great interest as important pollinating group, and some species are crop-pests or help to control them [26,27,28]. In relation to the hymenopterans, it is well-known that they play fundamental roles, outstanding is their role in the pollination process, their great agricultural implication, and their use as parasitoids for the pest control [29]. The order Lepidoptera is another large group involved in pollination and some butterfly families are of special interest due to their agricultural involvement, since the larvae of most species are phytophagous and thus being commonly pests in crops and in forest stands [27,28].

**Figure 7:**
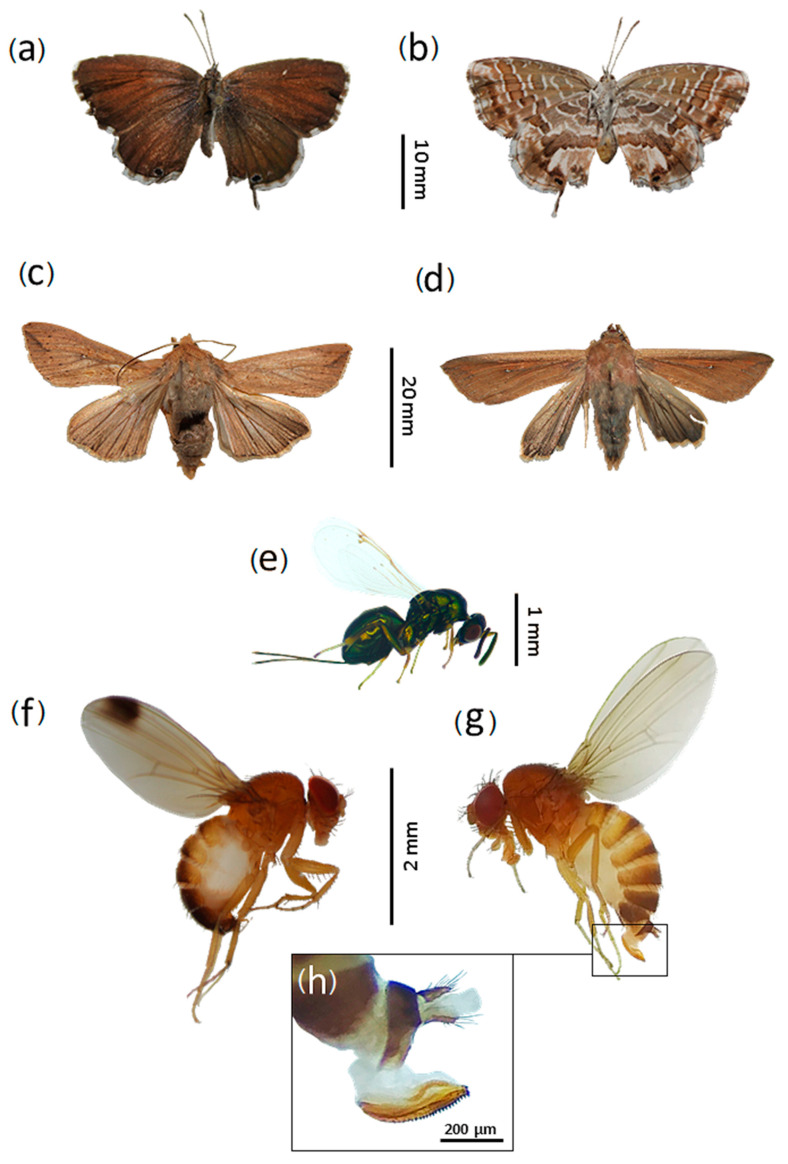
Exotic and invasive species captured in the studied *V. velutina* traps. *Cacyreus marshalli* (Lepidoptera: Lycaenidae) dorsal view (**a**); ventral view of the same (**b**); Male of *Mythimna unipuncta* (Lepidoptera: Noctuidae) (**c**); female of the same (**d**); female of *Torymus sinensis* (Hymenoptera: Torymidae) (**e**); male of *Drosophila suzukii* (Diptera: Drosophilidae) (**f**); female of the same (**g**); detail of the oviscapt of female *D. suzukii* (**h**).

Here, we have reported a specific richness of non-target species of insects in *V. velutina* traps of 74 taxa. Previous studies of non-target insects affected by *V. velutina* traps show similar trends to those reported here, the most affected groups being the orders Diptera, Hymenoptera and Lepidoptera [18,19]. However, in both studies a large number of ant specimens (Family Formicidae) were found, even exceeding the total number of dipterans [18], while in this work only two specimens were trapped. Among the identified species, four were exotic or non-indigenous to Spain, *C. marshalli* (Figure 7a,b), *M. unipuncta* (Figure 7c,d), *D. suzukii* (Figure 7f–h), and *T. sinensis* (Figure 7e), being the first three, in addition, invasive. *Drosophila suzukii* was the most captured species in studied *V. velutina* traps, with 4629 specimens caught and representing the 36.1% of the total catches (Table 1). *Drosophila suzukii* is an indigenous species from Asia (China, Japan, Korea, and Thailand) [30]. After its introduction to Spain in 2008, it caused serious damage to thin-skinned fruits such as cherries, blueberries, raspberries, blackberries and strawberries, both in cultured growing areas and in natural environments [30,31]. These damages are produced by its oviscapt morphology (Figure 7c), since it presents a series of denticles that allow *D. suzukii* to attack healthy fruits and not only those that are ripe or in decomposition process, as is usual in native *Drosophila* species [31,32]. This is especially relevant since *D. suzukii* may cause the ‘human intestinal myiasis’, due to the ingestion of apparently healthy fruits infected with larvae of this species [31,32]. The introduction of this species in Europe is related to the importation of fruit from East Asian countries [31]. The high abundance of this species during the summer may be related to the appearance of the fleshy fruits that it attacks. It is worth noting that *D. melanogaster*, its native congener, showed a completely inverse trend to *D. suzukii*, showing a low general abundance through the year and reaching its maximum in winter (Figure 6). *Cacyreus marshalli*, commonly known as geranium bronze, is a native species of Africa (Botswana, South Africa, Mozambique, Zimbabwe, and Lesotho) [33]. In its native range it frequents garden areas where they feed on Geraniaceae, a family of plants that includes genera such as *Geranium* and *Pelargonium* [34]. The first record of this species in Europe dated back to 1989 from the Balearic Islands, where it was reported as a plague of the ornamental geraniums (*Pelargonium*) [35]. However, shortly thereafter, it has been shown that, in the absence of the ornamental *Pelargonium* species, *C. marshalli* can attack native species of the genus *Geranium*, and furthermore they can outcompete with indigenous lycaenid species, such as *Aricia nicias* Meigen, 1830 and *Eumedonia eumedon* Esper, 1780 [34]. The presence of this species in the studied samples was very low, only four specimens were trapped (0.03% of the total). Finally, the true armyworm moth, *M. unipuncta*, it is an autochthonous species of North America (USA and Canada) [36]. This moth has great agricultural importance and is considered a pest species due to their damage in corn, oat, rice and other grass crops (caused by its larvae, known as soldier worms) [36,37].

## 5. Conclusions

The present work has constituted a pioneering study on the species-specific diversity of non-target species of the traps used to control the invasive *V. velutina* in northern Spain. These findings may be extended/compared to other parts of the Iberian Peninsula and to temperate regions of southern Europe (both with similar entomofauna). Specific entomofaunal identification studies, although laborious, are very informative and essential to mitigate the ecological repercussions of the control activities *V. velutina* may generate. Since, as it is well-known most of the non-target species play important ecological roles as pollinators and decomposers, among others. If we only considered the provided services/disservices by the non-target insect species found, *V. velutina* traps look very detrimental since they catch many species that play important ecological roles. However, on a broader view, most of the species are caught in very-low numbers, and if we look at the numeric contribution of individuals, the invasive species become dominant. The orders of insects most affected by the trapping of *V. velutina* were Diptera, Hymenoptera and Lepidoptera, being the invasive species *D. suzukii*, the most abundant species in the traps and representing 36.07% of the total catches. Concerning hymenopterans, ‘vespa-catch’ (veto-pharma) attractant showed to be selective for wasps of the genus *Vespa* (and *Vespula* to a lesser extent) since the capture of other hymenopteran species was practically anecdotal. Although the most affected hymenopteran by traps was the target species *V. velutina* (accounted for 2.23% of catches), the European species *V. crabro* was the following species, the second hornet species in the account, with an abundance in traps of 1.45%. As for the lepidoptera, the presence of the exotic species, *M. unipuncta*, which was found during the whole annual cycle, stands out due to its large size and by the fact that when these moths fall into the trap, they immediately swell and greatly increase their volume, and subsequently saturate the traps (reducing its effectiveness). Finally, the newly reported relationship between *V. velutina* and the ectoparasite mites of the genus *Varroa* needs to be studied further, since the Asian wasp could constitute a new dispersal vector of this parasite and its potential carried viruses.

## Figures and Tables

**Table 1:** Overall trapped species (total) and species per season. For each season, the number of collected specimens per trap and the percentage of trapped specimens per species total number of trapped insects are shown.

Order	Family	Species	Author	N. of Specimens (Spring)	% of Insects (Spring)	N. of Specimens (Summer)	% of Insects (Summer)	N. of Specimens (Autumn)	% of Insects (Autumn)	N. of Specimens (Winter)	% of Insects (Winter)	N. of Specimens (Total)	% of Insects (Total)
Diptera	Anisopodidae	*Sylvicola cinctus*	(Fabricius, 1787)	32	0.25%	70	0.55%	34	0.26%	71	0.55%	207	1.61%
Diptera	Anisopodidae	*Sylvicola fuscatus*	(Fabricius, 1775)	5	0.04%	15	0.12%	32	0.25%	1	0.01%	53	0.41%
Diptera	Anisopodidae	*Sylvicola punctatus*	(Fabricius, 1787)	72	0.56%	139	1.08%	118	0.92%	102	0.79%	431	3.36%
Diptera	Calliphoridae	*Bellardia viarum*	(Robineau-Desvoidy, 1830)	1	0.01%	0	0.00%	0	0.00%	0	0.00%	1	0.01%
Diptera	Calliphoridae	*Bellardia vulgaris*	(Robineau-Desvoidy, 1830)	1	0.01%	0	0.00%	0	0.00%	0	0.00%	1	0.01%
Diptera	Calliphoridae	*Calliphora vicina*	Robineau-Desvoidy, 1830	39	0.30%	122	0.95%	31	0.24%	123	0.96%	315	2.45%
Diptera	Calliphoridae	*Calliphora vomitoria*	(Linnaeus, 1758)	1	0.01%	7	0.05%	0	0.00%	13	0.10%	21	0.16%
Diptera	Calliphoridae	*Chrysomya albiceps*	(Wiedemann, 1819)	2	0.02%	3	0.02%	0	0.00%	2	0.02%	7	0.05%
Diptera	Calliphoridae	*Lucilia ampullacea*	Villenueve, 1922	1	0.01%	8	0.06%	6	0.05%	1	0.01%	16	0.12%
Diptera	Calliphoridae	*Lucilia caesar*	(Linnaeus, 1758)	189	1.47%	488	3.80%	63	0.49%	183	1.43%	923	7.19%
Diptera	Calliphoridae	*Lucilia illustris*	Meigen, 1826	4	0.03%	8	0.06%	3	0.02%	11	0.09%	26	0.20%
Diptera	Calliphoridae	*Morinia doronici*	(Scopoli, 1763)	1	0.01%	4	0.03%	1	0.01%	0	0.00%	6	0.05%
Diptera	Calliphoridae	*Onesia floralis*	Robineau-Desvoidy, 1830	0	0.00%	7	0.05%	0	0.00%	0	0.00%	7	0.05%
Diptera	Calliphoridae	*Pollenia griseotomentosa*	(Jacentkovsky, 1944)	6	0.05%	0	0.00%	0	0.00%	0	0.00%	6	0.05%
Diptera	Calliphoridae	*Pollenia labialis*	Robineau-Desvoidy, 1830	1	0.01%	0	0.00%	0	0.00%	0	0.00%	1	0.01%
Diptera	Calliphoridae	*Pollenia rudis*	(Fabricius, 1794)	49	0.38%	148	1.15%	33	0.26%	86	0.67%	316	2.46%
Diptera	Calliphoridae	*Stomorhina lunata*	(Fabricius, 1794)	15	0.12%	29	0.23%	0	0.00%	0	0.00%	44	0.34%
Diptera	Chloropidae	*Meromyza femorata*	Macquart, 1835	0	0.00%	3	0.02%	0	0.00%	0	0.00%	3	0.02%
Diptera	Chloropidae	*Thaumatomyia notata*	(Meigen, 1830)	5	0.04%	3	0.02%	9	0.07%	24	0.19%	41	0.32%
Diptera	Drosophilidae	*Drosophila melanogaster*	Meigen, 1830	9	0.07%	20	0.16%	22	0.17%	84	0.65%	135	1.05%
Diptera	Drosophilidae	*Drosophila suzukii*	(Matsumura, 1931)	676	5.27%	2629	20.48%	744	5.80%	580	4.52%	4629	36.07%
Diptera	Drosophilidae	*Gitona distigma*	Meigen, 1830	0	0.00%	3	0.02%	0	0.00%	2	0.02%	5	0.04%
Diptera	Fanniidae	*Fannia canicularis*	(Linnaeus, 1761)	1	0.01%	10	0.08%	42	0.33%	36	0.28%	89	0.69%
Diptera	Fanniidae	*Fannia* cf. *postica*	(Stein, 1895)	25	0.19%	4	0.03%	36	0.28%	50	0.39%	115	0.90%
Diptera	Lauxanidae	*Sapromyza opaca*	Becker, 1895	1	0.01%	0	0.00%	0	0.00%	1	0.01%	2	0.02%
Diptera	Muscidae	*Dasyphora albofasciata*	(Macquart in Webb & Berthelot, 1839)	18	0.14%	61	0.48%	1	0.01%	2	0.02%	82	0.64%
Diptera	Muscidae	*Mesembrina meridiana*	(Linnaeus, 1758)	2	0.02%	1	0.01%	1	0.01%	1	0.01%	5	0.04%
Diptera	Muscidae	*Musca autumnalis*	(De Geer, 1776)	1	0.01%	3	0.02%	10	0.08%	53	0.41%	67	0.52%
Diptera	Muscidae	*Musca domestica*	Linnaeus, 1758	9	0.07%	24	0.19%	8	0.06%	38	0.30%	79	0.62%
Diptera	Muscidae	*Musca tempestiva*	Fallén, 1817	0	0.00%	1	0.01%	0	0.00%	0	0.00%	1	0.01%
Diptera	Muscidae	*Muscina levida*	(Harris, 1780)	17	0.13%	44	0.34%	32	0.25%	37	0.29%	130	1.01%
Diptera	Muscidae	*Muscina pascuorum*	(Meigen, 1826)	6	0.05%	22	0.17%	1	0.01%	0	0.00%	29	0.23%
Diptera	Muscidae	*Muscina prolapsa*	(Harris, 1780)	48	0.37%	110	0.86%	11	0.09%	34	0.26%	203	1.58%
Diptera	Muscidae	*Muscina stabulans*	(Fallén, 1817)	18	0.14%	31	0.24%	0	0.00%	1	0.01%	50	0.39%
Diptera	Muscidae	*Mydaea scutellaris*	Robineau-Desvoidy, 1830	12	0.09%	46	0.36%	7	0.05%	2	0.02%	67	0.52%
Diptera	Muscidae	*Mydaea urbana*	(Meigen, 1826)	2	0.02%	3	0.02%	3	0.02%	0	0.00%	8	0.06%
Diptera	Muscidae	*Neomyia cornicina*	(Fabricius, 1781)	3	0.02%	4	0.03%	1	0.01%	3	0.02%	11	0.09%
Diptera	Muscidae	*Phaonia bitincta*	Róndani, 186	53	0.41%	87	0.68%	2	0.02%	24	0.19%	166	1.29%
Diptera	Muscidae	*Phaonia pallida*	(Fabricius, 1787)	100	0.78%	202	1.57%	0	0.00%	0	0.00%	302	2.35%
Diptera	Muscidae	*Polietes lardarius*	Fabricius, 1781	3	0.02%	0	0.00%	8	0.06%	152	1.18%	163	1.27%
Diptera	Muscidae	*Pyrellia vivida*	Robineau-Desvoidy, 1830	0	0.00%	0	0.00%	2	0.02%	0	0.00%	2	0.02%
Diptera	Phoridae	*Phoridae* sp.		6	0.05%	27	0.21%	22	0.17%	59	0.46%	114	0.89%
Diptera	Sarcophagidae	*Blaesoxipha* cf. *rossica*	Villeneuve, 1912	1	0.01%	1	0.01%	0	0.00%	0	0.00%	2	0.02%
Diptera	Sarcophagidae	*Sarcophaga* aff. *argyrostoma*	(Robineau–Desvoidy, 1830)	6	0.05%	10	0.08%	0	0.00%	2	0.02%	18	0.14%
Diptera	Sarcophagidae	*Sarcophaga* cf. *haemorrhoidalis*	Böttcher, 1913	9	0.07%	20	0.16%	6	0.05%	2	0.02%	37	0.29%
Diptera	Sarcophagidae	*Sarcophagidae* sp.		19	0.15%	44	0.34%	16	0.12%	16	0.12%	95	0.74%
Diptera	Scathophagidae	*Scathophaga stercolaria*	(Linnaeus, 1758)	3	0.02%	0	0.00%	11	0.09%	61	0.48%	75	0.58%
Diptera	Scatopsidae	*Reichertella pulicaria*	(Loew, 1846)	141	1.10%	229	1.78%	115	0.90%	663	5.17%	1148	8.94%
Diptera	Sciaridae	*Sciaridae* sp.		209	1.63%	518	4.04%	523	4.07%	436	3.40%	1686	13.14%
Diptera	Sirphidae	*Myatropa florea*	(Linnaeus, 1758)	0	0.00%	0	0.00%	1	0.01%	0	0.00%	1	0.01%
Diptera	Tabanidae	*Tabanus sudeticus*	Zeller, 1842	0	0.00%	1	0.01%	0	0.00%	0	0.00%	1	0.01%
Diptera	Trichoceridae	*Trichocera annulata*	Meigen, 1818	0	0.00%	0	0.00%	2	0.02%	0	0.00%	2	0.02%
Diptera	Ulidiidae	*Physiphora alceae*	(Preyssler, 1791)	4	0.03%	18	0.14%	2	0.02%	1	0.01%	25	0.19%
Hymenoptera	Torymidae	*Torymus sinensis*	(Muller, 1764)	0	0.00%	0	0.00%	0	0.00%	5	0.04%	5	0.04%
Hymenoptera	Torymidae	*Torymus auratus*	Kamijo, 1892	0	0.00%	0	0.00%	0	0.00%	8	0.06%	8	0.06%
Hymenoptera	Apidae	*Apis mellifera*	(Linnaeus, 1758)	1	0.01%	0	0.00%	0	0.00%	0	0.00%	1	0.01%
Hymenoptera	Apidae	*Bombus terrestris lusitanicus*	Krüger, 1956	1	0.01%	0	0.00%	0	0.00%	0	0.00%	1	0.01%
Hymenoptera	Formicidae	*Plagiolepsis* sp.		1	0.01%	0	0.00%	0	0.00%	0	0.00%	1	0.01%
Hymenoptera	Formicidae	*Solenopsis* sp.		0	0.00%	0	0.00%	1	0.01%	0	0.00%	1	0.01%
Hymenoptera	Vespidae	*Vespa crabro*	Linnaeus, 1758	25	0.19%	9	0.07%	136	1.06%	16	0.12%	186	1.45%
Hymenoptera	Vespidae	*Vespa velutina*	Lepeletier, 1836	81	0.63%	6	0.05%	112	0.87%	87	0.68%	286	2.23%
Hymenoptera	Vespidae	*Vespula germanica*	(Fabricius, 1793)	2	0.02%	0	0.00%	5	0.04%	0	0.00%	7	0.05%
Hymenoptera	Vespidae	*Vespula vulgaris*	(Linnaeus, 1758)	3	0.02%	0	0.00%	2	0.02%	0	0.00%	5	0.04%
Lepidoptera	Uncertain	*Lepidoptera* sp. 1		17	0.13%	31	0.24%	0	0.00%	0	0.00%	48	0.37%
Lepidoptera	Uncertain	*Lepidoptera* sp. 2		0	0.00%	1	0.01%	0	0.00%	0	0.00%	1	0.01%
Lepidoptera	Licenidae	*Cacyreus marshalli*	Butler, 1897	0	0.00%	1	0.01%	3	0.02%	0	0.00%	4	0.03%
Lepidoptera	Noctuidae	*Mythimna unipuncta*	(Haworth, 1809)	62	0.48%	92	0.72%	94	0.73%	46	0.36%	294	2.29%
Lepidoptera	Nymphalidae	*Pararge aegeria*	(Linnaeus, 1758)	2	0.02%	0	0.00%	0	0.00%	0	0.00%	2	0.02%
Blattodea	Blattidae	*Blatta orientalis*	Linnaeus, 1758	0	0.00%	0	0.00%	4	0.03%	0	0.00%	4	0.03%
Hemiptera	Pentatomidae	*Nezara viridula*	Linnaeus, 1758	1	0.01%	0	0.00%	0	0.00%	0	0.00%	1	0.01%
Mecoptera	Panorpidae	*Panorpa communis*	Linnaeus, 1758	0	0.00%	2	0.02%	1	0.01%	0	0.00%	3	0.02%
Neuroptera	Chrysopidae	*Pseudomellada clathratus*	(Schneider, 1845)	0	0.00%	1	0.01%	0	0.00%	0	0.00%	1	0.01%
Neuroptera	Chrysopidae	*Pseudomellada flavifrons*	(Brauer, 1851)	1	0.01%	4	0.03%	0	0.00%	0	0.00%	5	0.04%
Neuroptera	Chrysopidae	*Pseudomellada marianus*	(Navás, 1905)	1	0.01%	1	0.01%	0	0.00%	0	0.00%	2	0.02%
				2024	15.77%	5375	41.88%	2317	18.05%	3114	24.26%	12,835	100%

**Table 2:** List of species found with the ecological service/disservice provided and their 2018status’ (exotic and invasive species are highlighted in red colour). PO: pollinator; DE: decomposer; ADV: animal disease vector; HDV: human disease vector; PC: pest control; AD: agriculture damage; PA: parasitoids; PR: predators; NA: not applicable.

Scientific Name	PO	DE	ADV	HDV	PC	AD	PA	PR	Status
*Sylvicola cinctus*		+							Native
*Sylvicola fuscatus*		+							Native
*Sylvicola punctatus*		+							Native
*Bellardia viarum*	+	+							Native
*Bellardia vulgaris*	+	+							Native
*Calliphora vicina*	+	+	+						Native
*Calliphora vomitoria*	+	+	+						Native
*Chrysomya albiceps*	+	+	+						Native
*Lucilia ampullacea*	+	+							Native
*Lucilia caesar*	+	+	+						Native
*Lucilia illustris*	+	+	+						Native
*Morinia doronici*	+	+							Native
*Onesia floralis*	+	+							Native
*Pollenia griseotomentosa*	+						+		Native
*Pollenia labialis*	+						+		Native
*Pollenia rudis*	+						+		Native
*Stomorhina lunata*	+	+			+				Native
*Meromyza femorata*		+							Native
*Thaumatomyia notata*	+	+			+			+	Native
*Drosophila melanogaster*		+							Native
*Drosophila suzukii*		+				+			Invasive
*Gitona distigma*		+							Native
*Fannia canicularis*		+							Native
*Fannia postica*		+							Native
*Sapromyza opaca*	+	+							Native
*Dasyphora albofasciata*	+	+							Native
*Mesembrina meridiana*	+	+						+	Native
*Musca autumnalis*	+	+	+	+					Native
*Musca domestica*	+	+	+	+					Native
*Musca tempestiva*	+	+	+	+					Native
*Muscina levida*	+	+	+	+				+	Native
*Muscina pascuorum*	+	+	+	+				+	Native
*Muscina prolapsa*	+	+	+	+				+	Native
*Muscina stabulans*	+	+	+	+				+	Native
*Mydaea Scutellaris*	+	+	+						Native
*Mydaea urbana*	+	+	+						Native
*Neomyia cornicina*	+	+							Native
*Phaonia bitincta*	+							+	Native
*Phaonia pallida*	+							+	Native
*Polietes lardarius*	+	+							Native
*Pyrellia vivida*	+	+							Native
*Phoridae* sp.1		+							NA
*Blaesoxipha* cf. *rossica*	+	+							Native
*Sarcophaga* aff. *argyrostoma*	+	+							Native
*Sarcophaga* cf. *haemorrhoidalis*	+	+							Native
*Sarcophagidae* sp.	+	+							NA
*Scathophaga stercolaria*	+	+			+			+	Native
*Reichertella pulicaria*		+							Native
*Sciaridae* sp.		+				+			NA
*Myatropa florea*	+	+							Native
*Tabanus sudeticus*	+	+						+	Native
*Trichocera annulata*									Native
*Physiphora alceae*		+							Native
*Torymus sinensis*					+		+		Exotic
*Torymus auratus*					+		+		Native
*Apis mellifera*	+								Native
*Bombus terrestris lusitanicus*	+								Native
*Plagiolepsis* sp.									Native
*Solenopsis* sp.									Native
*Vespa crabro*	+							+	Native
*Vespa velutina*	+							+	Invasive
*Vespula germanica*	+							+	Native
*Vespula vulgaris*	+							+	Native
*Lepidoptera* sp. 1	+								NA
*Lepidoptera* sp. 2	+								NA
*Cacyreus marshalli*	+					+			Invasive
*Mythimna unipuncta*	+					+			Invasive
*Pararge aegeria*	+								Native
*Blatta orientalis*		+	+						Native
*Nezara viridula*						+			Native
*Panorpa communis*		+						+	Native
*Pseudomallada clathratus*	+				+			+	Native
*Pseudomallada flavifrons*	+				+			+	Native
*Pseudomallada marianus*	+				+			+	Native

## Data Availability

All data generated or analysed during this study are included in this published article.

## Appendix A

**Table A1:** SIMPER analysis of Group A (summer) and Group B (spring, autumn and winter). The dominant 12 insect species contributing to the similarity/dissimilarity of each group are tabulated.

Cluster/Taxa	% Contribution
Group A similarity (66.55%)	
*Drosophila suzukii*	37.59
*Sciaridae* sp.	16.93
*Reichertella pulicaria*	7.52
*Lucilia caesar*	6.18
*Vespa velutina*	5.05
*Sylvicola punctatus*	4.94
*Mythimna unipuncta*	3.17
*Pollenia rudis*	2.32
*Calliphora vicina*	2.05
*Sylvicola cinctus*	1.99
*Fannia* cf. *postica*	1.73
*Muscina levida*	1.31
Group B (Less than 2 samples in group)	
Group A & B dissimilarity (66.55%)	
*Drosophila suzukii*	50.35
*Lucilia caesar*	8.83
*Reichertella pulicaria*	5.22
*Phaonia pallida*	4.28
*Sciaridae* sp.	3.49
*Pollenia rudis*	2.39
*Vespa velutina*	2.24
*Muscina prolapsa*	2.03
*Calliphora vicina*	1.55
*Phaonia bitincta*	1.55
*Dasyphora albofasciata*	1.38
*Vespa crabro*	1.31

## References

[1] Haxaire J., Bouguet J.P., Tamisier J.P. (2006). *Vespa velutina* Lepeletier, 1836, une redoutable nouveauté pour la faune de France (Hym., Vespidae). Bull. Soc. Entomol. Fr..

[2] Villemant C., Haxaire J., Streito J.C. (2006). The discovery of the Asian hornet *Vespa velutina* in France. (La découverte du frelon asiatique Vespa velutina, en France). Insectes.

[3] Caragata C.R., Montesinos J.L.V. (2020). Datos ambientales preliminares del avispón asiático (*Vespa velutina* Lepeletier, 1836) (Hymenoptera, Vespidae) en Asturias, España. Bol. R. Soc. Esp. Hist. Nat..

[4] Barbet-Massin M., Rome Q., Muller F., Perrard A., Villemant C., Jiguet F. (2013). Climate change increases the risk of invasion by the yellow-legged hornet. Biol. Conserv..

[5] Castro L., Pagola-Carte S. (2010). *Vespa velutina* Lepeletier, 1836 (Hymenoptera: Vespidae), recolectada en la Península Ibérica. Heteropterus Rev. Entomol..

[6] Rodríguez-Flores M.S., Seijo-Rodríguez A., Escuredo O., Seijo-Coello M.C. (2019). Spreading of *Vespa velutina* in northwestern Spain: Influence of elevation and meteorological factors and effect of bait trapping on target and non-target living organisms. J. Pest. Sci..

[7] Monceau K., Bonnard O., Thiéry D. (2014). *Vespa velutina*: A new invasive predator of honeybees in Europe. J. Pest Sci..

[8] Young J., Watt A., Nowicki P., Alard D., Clitherow J., Henle K., Johnson R., Laczko E., McCracken D., Matouch S. (2005). Towards sustainable land use: Identifying and managing the conflicts between human activities and biodiversity conservation in Europe. Biodivers. Conserv..

[9] Henle K., Alard D., Clitherow J., Cobb P., Firbank L., Kull T., McCracken D., Moritz R.F.A., Niemelä J., Rebane M. (2008). Identifying and managing the conflicts between agriculture and biodiversity conservation in Europe—A review. Agric. Ecosyst. Environ..

[10] Monceau K., Maher N., Bonnard O., Thiéry D. (2015). Evaluation of competition between a native and an invasive hornet species: Do seasonal phenologies overlap?. Bull. Entomol. Res..

[11] López S., González M., Goldarazena A. (2011). *Vespa velutina* lepeletier, 1836 (Hymenoptera: Vespidae): First records in Iberian Peninsula. Bull. OEPP.

[12] Haro L., Labadie M., Chanseau P., Cabot C., Blanc-Brisset I., Penouil F. (2010). Medical consequences of the Asian black hornet (*Vespa velutina*) invasion in Southwestern France. Toxicon.

[13] Arca M., Papachristoforou A., Mougel F., Rortais A., Monceau K., Bonnard O., Tardy P., Thiéry D., Silvain J.F., Arnold G. (2014). Defensive behaviour of *Apis mellifera* against *Vespa velutina* in France: Testing whether European honeybees can develop an effective collective defense against a new predator. Behav. Process..

[14] Budge G.E., Hodgetts J., Jones E.P., Ostojá-Starzewski J.C., Hall J., Tomkies V., Semmence N., Brown M., Wakefield M., Stainton K. (2017). The invasion, provenance and diversity of *Vespa velutina* Lepeletier (Hymenoptera: Vespidae) in Great Britain. PLoS ONE.

[15] Leza M., Miranda M.Á., Colomar V. (2018). First detection of *Vespa velutina nigrithorax* (Hymenoptera: Vespidae) in the Balearic Islands (Western Mediterranean): A challenging study case. Biol. Invasions.

[16] Lioy S., Manino A., Porporato M., Laurino D., Romano A., Capello M., Bertolino S. (2019). Establishing surveillance areas for tackling the invasion of *Vespa velutina* in outbreaks and over the border of its expanding range. NeoBiota.

[17] Laurino D., Lioy S., Carisio L., Manino A., Porporato M. (2020). *Vespa velutina*: An Alien Driver of HoneyBee Colony Losses. Diversity.

[18] Lioy S., Laurino D., Capello M., Romano A., Manino A., Porporato M. (2020). Effectiveness and Selectiveness of Traps and Baits for Catching the Invasive Hornet *Vespa velutina*. Insects.

[19] Rojas-Nossa S.V., Novoa N., Serrano A., Calviño-Cancela M. (2018). Performance of baited traps used as control tools for the invasive hornet *Vespa velutina* and their impact on non-target insects. Apidologie.

[20] Primack R.B., Silander J.A. (1975). Measuring the relative importance of different pollinators to plants. Nature.

[21] Klein A.M., Vaissiere B.E., Cane J.H., Steffan-Dewenter I., Cunningham S.A., Kremen C., Tscharntke T. (2007). Importance of pollinators in changing landscapes for world crops. Proc. R. Soc. B.

[22] Bray J.R., Curtis J.T. (1957). An ordination of the upland forest communities of Southern Wisconsin. Ecol. Monogr..

[23] Anderson D.L., Trueman J.W.H. (2000). *Varroa jacobsoni* (Acari: Varroidae) is more than one species. Exp. Appl. Acarol..

[24] Jelinski M. (1990). Roztocz *Varroa jacobsoni* Oudemans, 1904 na larwach osy pospolitej Vespa (Paravespula) vulgaris L.. Wiad Parazytol..

[25] Mordecai G.J., Brettell L.E., Pachori P., Villalobos E.M., Martin S.J., Jones I.M., Schroeder D.C. (2016). Moku virus; a new Iflavirus found in wasps, honeybees and *Varroa*. Sci. Rep..

[26] Gállego B.J. (2006). Manual de Parasitología: Morfología y Biología de Los Parásitos de Interés Sanitario.

[27] Balachowsky A.S., Masson e.C. (1972). Entomologie Appliquée à 1’Agriculture. Tome II. Lepidópteros, Deuxième Volume.

[28] Scoble M.J. (1992). The Lepidoptera. Form, Function and Diversity.

[29] Southwick E.E., Southwick L. (1992). Estimating the economic value of honeybees (Hymenoptera: Apidae) as agricultural pollinators in the United States. J. Econ. Entomol..

[30] Calabria G., Máca J., Bächil G., Serra L., Pascual M. (2012). First records of the potencial pest species *Drosophila suzukii* (Diptera: Drosophilidae) in Europe. J. Appl. Entomol..

[31] Bieńkowski A.O., Orlova-Bienkowskaja M.J. (2020). Invasive Agricultural Pest *Drosophila suzukii* (Diptera, Drosophilidae) Appeared in the Russian Caucasus. Insects.

[32] Fiel R.A., Narganes A.G., Argüelles M.B. (2014). Incidencia de “*Drosophila suzukii*” en cultivos de arándano y frambuesa en Asturias. Phytoma España.

[33] Paradiso F., Martelli F., Cerrato C., Ghidotti S., Ramona V., Canterino S., Ferracini C., Bonelli S. (2019). From Africa to the Alps: Risk assessment on an invasion by *Cacyreus marshalli* (Butler, 1898). J. Insect. Conserv..

[34] Quacchia A., Ferracini C., Bonelli S., Balletto E., Alma A. (2008). Can the Geranium Bronze, *Cacyreus marshalli*, become a threat for European biodiversity?. Biodivers. Conserv..

[35] Eitschberger U., Stamer P. (1990). *Cacyreus marshalli* Butler, 1898, Eine neue Tagfalterart für sie Europaïsche Fauna. Lepidoptera, Lycaenidae). Atalanta.

[36] Brou V.A., Brou C.D. (2020). *Mythimna unipuncta* (Haworth, 1809) (Lepidoptera: Noctuidae) in Louisiana. South. Lepid. News.

[37] Hill D.S. (1983). Agricultural Insect Pest of the Tropics and Their Control.

